# Supplementary material for: Message Source Credibility and E-Cigarette Harm Perceptions among Young Adults
Source: Int J Environ Res Public Health. 2022 Jul 26;19(15):9123. doi: 10.3390/ijerph19159123 (PMC9329714; doi:10.3390/ijerph19159123)
Supplement: Supplementary file 1 [file ijerph-19-09123-s001.zip › ijerph-1798005-supplementary.pdf]

What is an Electronic Vapor Product?

This message was written by a leading expert in tobacco research at US Food and Drug Administration (FDA)

An electronic vapor product (EVP) is sometimes called an e-cigarette, vape pen, e-hookah, or mod. All of these names refer to EVP. EVP can contain high amounts of nicotine, which is a highly addictive drug that causes addiction. All nicotine products, including cartridge-based and disposable vapes, can expose users' brain to nicotine and harm your health. This can disrupt brain development and cause long-term effects on attention, learning, and memory, and promote addiction to nicotine. Not all EVPs contain nicotine, but most do. And regardless of whether they have nicotine, all EVPs can contain toxic chemicals.

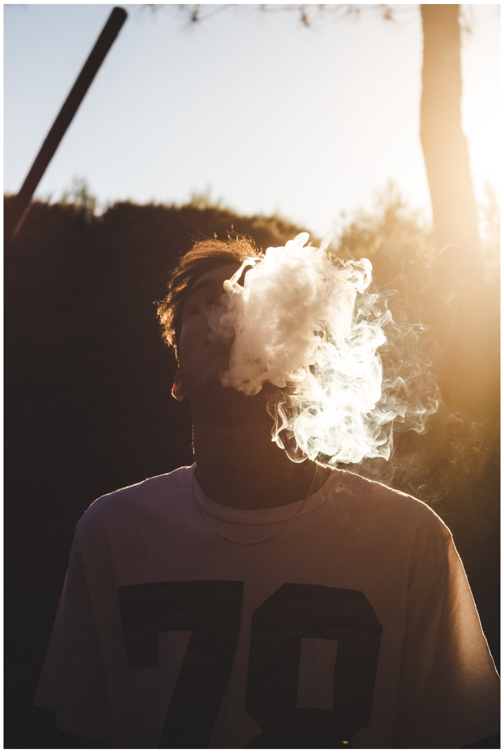

EVPs typically have liquid that is heated to create an aerosol, which the user inhales. It contains cancer causing toxicants. EVP liquids (e-liquids) typically consist of toxicants, nicotine, flavorings, and other chemicals. E-liquids are made primarily from chemicals that is known to cause lung damage and cancer.

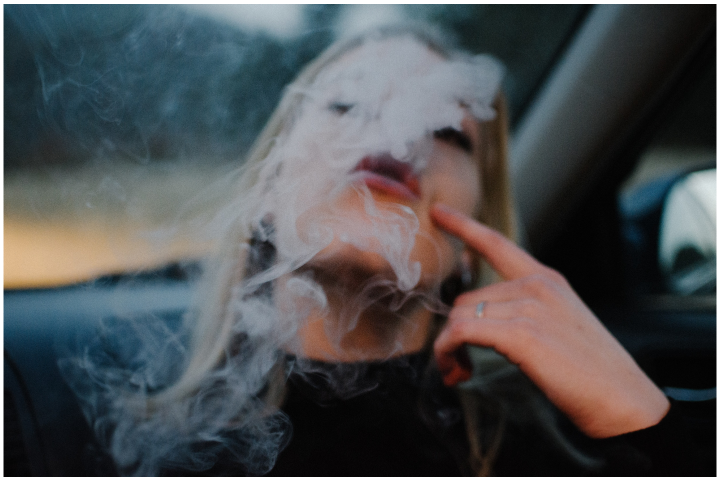

Using EVP can also expose people to metal particles that are known to be toxic to lungs, such as chromium, nickel, and lead. Some users may also experience coughing and wheezing, vomiting, nausea, headache, and dizziness.

EVPs have not been approved to help people quit smoking. The safest option for your health is to be completely tobacco-free, since nicotine in any form can be disruptive to brain development.

What is a Vape?

This message was written by a young adult just like you.

You might have heard people call vape by different names – e-cigarette, vape pen, e-hookah, or mod. All of them are electronic tobacco product. Vapes can have high amounts of nicotine, which is a highly addictive drug that causes addiction. All nicotine products, including refillable and disposable vapes, can affect your brain from nicotine and damage your health. This can mess with your brain development and cause long-term effects on your ability to pay attention, learn, and remember, and get addicted to nicotine. Not all vapes have nicotine, but most do. And regardless of whether they have nicotine, all vapes can have toxic chemicals.

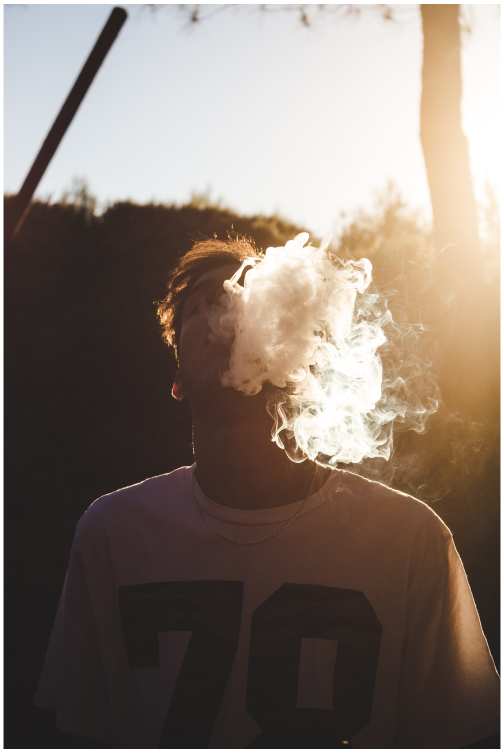

Vapes typically have liquid that is heated to create smoke that you breathe in. It has chemicals that can cause cancer. Vape liquids (e-liquids) have a variety of artificial substances, chemicals, nicotine, and flavorings. They make E-liquids from toxic alcohol-based liquid that breaks down into harmful chemicals that can destroy your lung and cause cancer.

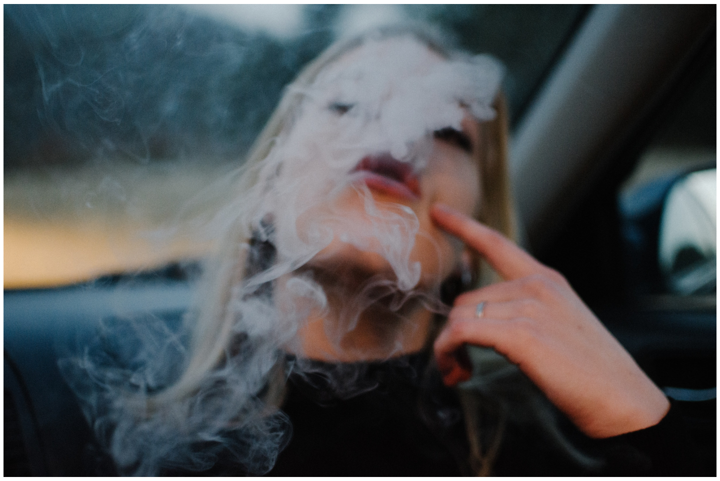

When you vape, you also breathe in toxic metal particles that can cause lung diseases. You can also start coughing and wheezing, vomiting, getting nauseous, headaches and dizzy.

Vapes have not been approved to help people quit smoking. The safest option for your health is to be completely tobacco-free because harmful substance from vaping can harm your brain development.
